# Selenium nanoparticle inclusion in broiler diets for enhancing sustainable production and health

**DOI:** 10.1038/s41598-024-67399-7

**Published:** 2024-08-09

**Authors:** Fatma S. O. Elkhateeb, Abdallah A. Ghazalah, Jayant Lohakare, Ahmed A. A. Abdel-Wareth

**Affiliations:** 1 Department of Animal and Poultry Production, Faculty of Agriculture, South Valley University, Qena, 83523 Egypt; 2 Department of Animal Production, Faculty of Agriculture, Cairo University, Giza, 12613 Egypt; 3 Poultry Center, Cooperative Agricultural Research Center, Prairie View A&M University, Prairie View, TX 77446 USA

**Keywords:** Antioxidant, Blood biochemistry, Broilers, Nanoparticles, Selenium retention, Biochemistry, Physiology

## Abstract

This study aimed to evaluate the effects of dietary supplementation of nanoparticles of Selenium (Nano-Se) on productive performance, nutrient digestibility, carcass criteria, selenium retention, blood biochemistry, and histopathological examination of broiler chicken. A total of 192 1-day-old male broiler chickens (Cobb 500) were randomly assigned to one of four treatment diets, with each diet given to six replicates of eight chicks. The birds were randomly assigned to one of four treatment groups, each of which included Nano-Se at levels of 0, 0.2, 0.3, or 0.4 mg/kg. The feeding experiment lasted 35 days. Nano-Se addition to broiler diets at 0.2 and 0.3 mg/kg enhanced body weight and body weight gain linearly compared to the control diet and 0.4 mg/kg. The apparent digestibility coefficient of ether extracts linearly increased with increasing Nano-Se levels up to 0.4 mg/kg. Increasing Nano-Se decreased serum cholesterol, triglycerides, alanine aminotransaminase, aspartate aminotransaminase, and creatinine in broiler chickens. Also, serum antioxidants showed a significant increase with increasing Nano-Se levels. As Nano-Se levels were supplemented, improvements in cooking loss, water-holding capacity, and antioxidants were observed as compared to the control. Additionally, a noticeable improvement in meat quality was observed regarding the obtained meat characters. It was preferred to use low doses of Nano-Se (0.3 mg/kg), as tissue retention of Se for both meat and liver was more comparable to the control. In conclusion, nutritional supplementation with Nano-Se increased growth performance, nutrient digestibility, selenium retention, meat quality, blood biochemistry, histological indices, and antioxidant activity of broiler chickens. Overall, the best performance of broilers was observed with Nano-Se supplementation at 0.3 mg/kg, highlighting its potential as a novel supplement for broiler diets.

## Introduction

Poultry meat, which includes chicken, turkey, duck, and other birds raised for meat production, compares favorably to other types of meat in terms of nutritional value. Broiler chickens, with their efficient growth, cost-effectiveness, and nutrient-rich meat, play a vital role in human nutrition and contribute to overall health^1^. Increasing requests for poultry meat as the consumers demand and for improving animal welfare has directed producers towards working at disease minimization to improve production^2^. Selenium plays critical roles in antioxidant defense, immune function, reproductive health, thyroid function, and muscle health in broiler nutrition^3,4^. Supplementing broiler diets with selenium helps meet their nutritional requirements and supports overall health, performance, and productivity^5^. Selenium (Se) is an essential trace element required for various physiological functions in animals, including broiler chickens^6,7^. Its deficiency can lead to impaired growth, compromised immune function, and increased susceptibility to diseases^8^. Antinutritional factors present in broiler diets, including phytate, oxalates, tannins, high sulfur content, and mycotoxins, can impede the absorption of selenium from feed ingredients. Addressing this challenge, Nano-Se, characterized by its superior bioavailability, presents a promising solution. Nanoparticles have a larger surface area compared to their bulk counterparts, which facilitates better absorption in the gastrointestinal tract of poultry. This means that the Nano-Se are absorbed more efficiently, leading to improved utilization by the birds. Furthermore, the application of nanoparticles of trace elements in poultry nutrition offers several advantages compared to other sources, including increased bioavailability, enhanced nutrient utilization, reduced environmental impact, precision nutrition, improved animal health and performance, and cost-effectiveness^9,10^. Nanoparticle of selenium (Nano-Se) supplementation has the potential to reduce selenium waste in the environment when compared to traditional selenium sources, and Nano-Se can be used more efficiently by broilers, resulting in lower selenium excretion in manure^11–13^. The significant biological activity of Nano-Se^14^ includes protection against DNA oxidation^15^ and anti-hydroxyl radical characteristics^16^, as the surface area-to-volume ratio increases with decreasing particle size. Further research by Zhang and coworkers^14^ showed that Nano-Se was more successful than selenite, selenomethionine, and methyl-selenocysteine^9,11^ at upregulating selenoenzymes in mice and rats and was also less hazardous^17^. Some studies have consistently pinpointed the ideal Se concentration for broiler supplementation to fall within the range of 0.1 to 0.6 mg/kg^9,18–20^. In guaranteeing adequate Se intake in broiler diets, a practical strategy is the integration of Nano-Se. Noteworthy for its exceptional bioavailability, minimal toxicity, and distinctive attributes, Nano-Se allows for lower dosage administration while delivering superior outcomes compared to conventional Se sources. Consequently, this research aims to assess the impact of novel dietary Nano-Se supplementation on broiler chicken performance, nutrient utilization, carcass quality, Se retention, blood biochemistry, and histopathological features.

## Materials and methods

### Diets and experimental design

The Department of Animal and Poultry Production, Faculty of Agriculture, South Valley University, Qena, Egypt, housed the birds in its research farm. The South Valley University Department of Animal and Poultry Production’s Committee of Ethics authorized the experiment protocol (SVU-AGRI-8-2020), the chicks were cared for throughout the trial according to the guidelines for the treatment of experimental animals and all methods were performed in accordance with the relevant guidelines and regulations. The study was carried out in compliance with the ARRIVE guidelines (https://arriveguidelines.org). A total of 192 1-day-old male broiler chickens (Cobb 500) were randomly assigned to one of four treatment diets, each of which included Nano-Se at levels of 0, 0.2, 0.3, or 0.4 mg/kg, with each diet given to six replicates of eight chicks. The feeding experiment lasted 35 days. Nano-Se was added to one kg of the basal diet with the required concentration and mixed well into the total amount before feeding to the corresponding group. Diets were offered in mash form. To meet the dietary requirements, the diets were created by guidelines for male Cobb 500 broilers (Table 1). Throughout the trial, all of the chicks' feeding and water needs were provided. The trial period consisted of three unique phases: the starting period, which lasted from 1 to 12 days, the grower period, which lasted from 13 to 23 days, and the finisher period, which lasted from 24 to 35 days. Measured floor pens were used to allocate the treatment groups according to class. Every chick was housed in wire-floored cages measuring 120 × 120 × 60 m^3^, situated in a climate-controlled chamber with constant lighting and unlimited access to feed and water. Initially, the ambient temperature was set at 32 °C for the first 3 days. Subsequently, it gradually decreased by 3 °C per week until reaching 23 °C, where it remained constant. The relative humidity was maintained between 50 and 70%. During the initial 3 days, continuous lighting was provided, followed by a 23-h light schedule for the remainder of the 5-week feeding experiment. Throughout the study, the birds had unrestricted access to both feed and water. The same environmental, sanitary, and management settings were applied to broiler housing throughout the five-week experimental period. There were no dead birds in our experiment is notable.

**Table 1:** Ingredients and nutrient composition of broiler starter, grower, and finisher diet.

Ingredients %	Starter (1–12 day)	Grower (13–23 day)	Finisher (24–35 day)
Yellow corn	52.02	55.74	60.86
Soy bean meal 44%	35.21	31.16	25.73
Corn gluten meal 60%	5.00	5.00	5.00
Soy bean oil	3.20	4.06	4.59
Limestone	1.73	1.49	1.40
Mono calcium phosphate	1.43	1.26	1.12
Vit. and minerals premix*	0.30	0.30	0.30
dl-methionine	0.28	0.23	0.20
l-Lysine HCL	0.31	0.24	0.27
Sodium chloride (salt)	0.32	0.32	0.33
Sodium bicarbonate	0.10	0.10	0.10
Choline chloride 60%	0.10	0.10	0.10
Total	100	100	100
Calculated analysis			
Crude protein%	23.00	21.50	19.00
Metabolizable energy (kcal/kg)	3000	3100	3200
Crude fiber%	3.85	3.65	3.37
Crude fat%	5.81	6.75	7.39
Lysine%	1.45	1.29	1.16
Methionine	0.69	0.51	0.56
Methionine + cysteine%	1.06	0.99	0.91
Calcium%	0.98	0.87	0.89
Available phosphorus%	0.45	0.44	0.41
Sodium%	0.17	0.17	0.17
Selenium, mg/kg	0.136	0.135	0.134

*Supplied per kg diet, biotin (50 mg), pantothenic acid (10,000 mg), folic acid (1000 mg), nicotinic acid (30,000 mg), A (1900 IU), K3 (1000 mg), B1 (1000 mg), B2 (5000 mg), B6 (1500 mg), and B12 (0.046 mg) in addition to D3 (1300 IU), E (10,000 mg), and BHT (10,000 mg) and includes 60 mg of Mn, 50 mg of Zn, 0.1 mg of Se, 4 mg of Cu, 3 mg of I, and 0.1 mg of Co.

### Nano-Se preparation

**Figure 1:**
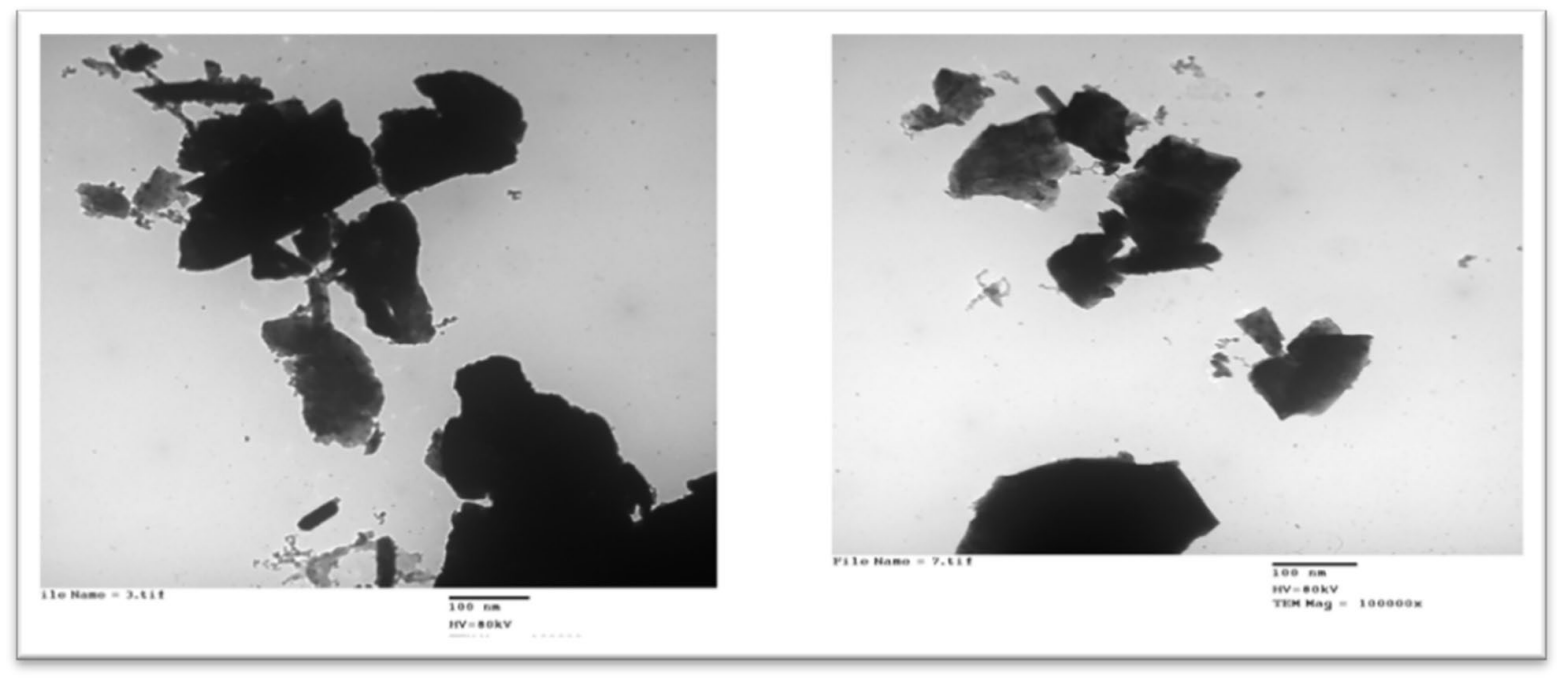
Selenium nanoparticles (Nano-Se); the transmission electron microscope (TEM) (**a**) and scanning electron microscope (SEM) (**b**) have dimensions of 1–100 nm, HV = 80 kv, and TEM Mag = 100,000.

Nano red elemental selenium (Nano-Se) can be produced by reducing selenite in an atmosphere that contains bovine serum albumin (BSA) using the redox system of selenite and glutathione (GSH). The concentration of BSA in the preparation solution affects the diameters of the nanoparticles. Four milliliters of 25 mM GSH containing 40 mg of BSA were mixed with one milliliter of 25 mM sodium selenite to create the Nano-Se. Smaller Nano-Se particles are produced by higher BSA concentrations. The product was a pink powder with a median size of 80 nm and a measurement of Se 10–100 nm. To get the required amount for diet formulation, the Nano-Se concentration was diluted to a manageable level using limestone as a carrier before being mixed with the feed. Transmission and scanning electron microscopy (TEM and SEM) were used to assess the structural morphology of the particles (Fig. 1).

### Growth performance parameters

Digital balance was used to weigh the birds in each replicate at the beginning and end of each feeding period. The difference between feed that was delivered and consumed after each feeding period was used to compute feed intake (FI). These data were used to determine body weight (BW), body weight gain (BWG), feed intake (FI), and feed conversion ratio (FCR). Throughout the research phase, the birds' death was also tracked every day.

### Digestibility trial

At the end of the experimental period, one healthy male chick was selected per replicate and kept in a metabolic cage for the digestion trial. The selected birds undergo an acclimation period of 4 days. Afterward, the birds undergo a short fasting period of 12 h before being subjected to feed intake measurements and fecal sample collections for four days. During these last 4 days of the study, the birds were given the mash experimental meal daily at the same time and were always given free access to clean drinking water. Excrement and feed residues were collected every day and weighed, rounding to the nearest 5 g, on an analytical scale. The excreta were frozen at − 10 °C until they were suitable for chemical analysis. Samples were defrosted, ground, and then partially dried at 60 °C for 48 h before analysis. The apparent digestibility coefficient (ADC) of the nutrients was calculated using the following formula: ADC [%] = [(t − f)/t] 100. Here, t denotes the amount of nutrients ingested during the collection time [g] and f denotes the amount of nutrients ejected [g].

### Chemical analysis

The diet and excreta samples were analyzed in the laboratories of the South Valley University Faculty of Agriculture using the following AOAC methods^21^: oven drying (method number 930.15), incineration (method number 942.05), crude protein (CP) by Kjeldahl (method number 984.13), and Soxhlet fat analysis (method number 920.39) for ether extract (EE). On the other hand, the Weende method (method 978.10) was used in the Cairo University, Faculty of Agriculture laboratories to determine crude fiber (CF). A global estimate of protein digestibility obtained by using the method published by Terpstra and de Hart^22^ which consists in chemically separating urinary nitrogen from undigested protein nitrogen, both of which are eliminated at the cloaca. Uric acid is solubilised and then undigested proteins are precipitated using lead acetate. The protein content is measured by the Kjeldhal method number 984.13.

### Blood biochemical assay

In summary, six birds from each replication (a total of 36 chicks per treatment) were randomly chosen for blood collection after the trial, which lasted 35 days. To reduce the effects of circadian fluctuations on the measured plasma parameters, blood was drawn early in the morning during the slaughter process. Centrifuge tubes were kept dry and clean for storing blood samples. The serum was centrifuged for 10 min at 3000 rpm at room temperature after it had spontaneously separated. The serum was collected in tubes and stored at − 20 °C for additional analysis. Triglycerides, glucose, total cholesterol, kidney and liver function which included creatinine, urea, alanine aminotransferase (ALT), aspartate aminotransferase (AST), and antioxidant status were among the blood parameters that were investigated in this study. In the investigated serum samples, the activities of the enzymes ALT, AST, uric acid, creatinine, and total cholesterol were measured using the spectrophotometric method (RAL, Barcelona, Spain) and Bioanalytica test kits (Bioanalitika doo, Beograd, Serbia)^23^. Antioxidant indices are determined by measuring the levels of glutathione peroxidase (GSH-Px), malondialdehyde (MDA), and total antioxidant capacity (T-AOC). Blood samples were used to measure the levels of GSH using the UV method^24^, while colorimetric methods were used to measure the levels of MDA, T-AOC, and the ability to inhibit the hydroxyl radical^25–27^.

### Carcass measurements

A total of 36 birds (six birds per replication, representing the pen) were used at random for each treatment, and they were weighed, slaughtered, and plucked. To ascertain the dressed weight, the remaining body parts were weighed following the removal of the neck, head, shanks, viscera, digestive tract, spleen, liver, gizzard, heart, and belly fat. Calculations were used to determine the dressing, breast, drumstick, and thigh proportions to the live weight. The percentages of abdominal fat, heart, liver, spleen, and empty gizzard were computed.

### Meat quality

After homogenizing 5 g of raw muscles with iodoacetate, the ultimate pH (pHu) of the meat was measured 24 h after the death using a Knick digital pH meter (Broadly Corp., Santa Ana, CA, USA)^28^. An electric oven preheated to 200 °C was used to cook muscle samples weighing approximately 5 g in an open aluminum pan for 15 min, or until the interior temperature reached 80 °C^29^. The quantity of cooking loss was measured by calculating the weight difference between the raw and cooked samples, expressed as a percentage of the raw sample after they were cooled for 30 min at 15 °C. The Water Holding Capacity (WHC) was determined by centrifuging 5 g of muscle that had been placed on tissue paper within a tube for 4 min at 1.500×*g*^30^. After centrifugation, the samples were dried for a whole night at 70 °C to determine how much water remained. To calculate the WHC, the weight after centrifugation minus the weight after drying was multiplied by 100 and the starting weight.

### Sampling selenium in tissues

In order to analyze the selenium content of tissues, 0.1 g of meat muscle or liver was weighed and then 8 mL of HNO3 was added to a digestion tube. Following mixing, the fluid was broken down in a microwave digestion machine. Deionized water was added to the solution to create a volume of 10 mL after part of the acid was removed using an adjustable electric heating plate set at 160 °C, leaving 1 mL of solution. The amount of selenium in the solution was ascertained [Agilent Technologies, Santa Clara, CA, used an Agilent 7500 series] inductively coupled plasma-mass spectrometer^31^.

### Histopathological examination

In the Faculty of Veterinary Medicine laboratories, at Cairo University, Tissue specimens of the intestine and liver were fixed in 10% neutral buffered formalin. The tissues were then processed by paraffin embedding technique, sectioned into 4 µm thick tissue sections, and stained by hematoxylin and eosin stain. A light microscope with a digital camera was used in the examination and photography of tissue.

### Statistical analysis

Using a completely randomized design and SAS 9.2’s general linear model (GLM) method, the statistical analysis was carried out^32^. The dosage of Nano-Se supplementation was a single element in the model. The experimental units used in performance parameters were pens. The birds were used as experimental units in other parameters. The linear and quadratic impacts of the increasing inclusion levels were ascertained using orthogonal polynomial contrasts, and the means were compared using Duncan's multiple range test. The threshold for significance was set at P < 0.05; P-values below 0.001 are not stated as the true value, but rather as “< 0.001”.

### Ethics approval and consent to participate

The Department of Animal and Poultry Production, Faculty of Agriculture, South Valley University’s Ethical Committee approved the current study (SVU-AGRI-8-2020), and all methods were performed in accordance with the relevant guidelines and regulations.

## Results

### Growth performance

The effects of supplementation of Nano-Se at 0, 0.2, 0.3, and 0.4 mg/kg in the broiler diets on growth performances are presented in Table 2. The results revealed that supplementations of Nano-Se linearly increased the BW of broilers at 12 days of age but supplementations of Nano-Se linearly and quadratically increased the BW of broilers at 24 and 35 days of age. Likewise, the BWG of broilers was significantly increased with increasing levels of Nano-Se during the whole period. Also, the FCR was significantly improved with increasing supplementations of Nano-Se to broiler diets during the periods of starter (1–12 days), finisher period (24–35 days), and whole period (1–35 days). Also, it deserves to be mentioned that the FI showed a linearly significant increase upon supplementation with 0.3 mg/kg at 13–24 and 1–35 days of age. The highest feed intake of broilers was observed with Nano-Se supplementation at 0.3 mg/kg.

**Table 2:** Effects of Nano-Se on growth performance of broiler chickens.

Items	Se-NPs levels mg/kg				SEM	p-value	
	0	0.2	0.3	0.4		Linear	Quadratic
Body weight, g							
1 day	43.25	42.39	43.50	42.18	0.837	0.584	0.787
12 days	355.4^b^	424.3^a^	411.1^a^	423.7^a^	18.17	0.028	0.136
24 days	1060^b^	1200^a^	1247^a^	1217^a^	25.69	0.002	0.003
35 days	1862^c^	2142^b^	2285^a^	2260^a^	58.25	0.001	0.016
Body weight gain, g							
1–12 days	312.1^b^	381.9^a^	367.6^a^	381.5^a^	18.05	0.026	0.137
13–24 days	705.4^b^	771.8^a^	836.1^a^	794.1^a^	23.62	0.005	0.032
24–35 days	801.7^c^	941.43^b^	1038^a^	1042^a^	59.43	0.005	0.268
1–35 days	1819^c^	2099^b^	2242^a^	2218^a^	57.95	0.001	0.017
Feed intake, g							
1–12 days	466.7	485.3	501.3	470.3	21.74	0.784	0.267
13–24 days	1127^c^	1101^c^	1303^a^	1184^b^	38.35	0.042	0.240
24–35 days	1467	1505	1601	1527	76.00	0.424	0.469
1–35 days	3061^c^	3092^c^	3406^a^	3182^b^	86.79	0.039	0.158
Feed conversion ratio							
1–12 days	1.499^a^	1.274^c^	1.368^b^	1.244^c^	0.056	0.014	0.045
13–24 days	1.613^a^	1.434^c^	1.561^b^	1.495^c^	0.067	0.458	0.052
24–35 days	1.838^a^	1.609^b^	1.569^b^	1.469^c^	0.068	0.001	0.057
1–35 days	1.683^a^	1.476^c^	1.519^b^	1.433^c^	0.039	0.008	0.064

*SEM* standard error of the means.

^a,b^Means within a row with different superscripts are significantly different (p < 0.05).

### Coefficient of total tract apparent digestibility

Table 3 displays the impact of Nano-Se levels on the nutritional digestibility of broiler chickens. The apparent digestibility coefficient of EE in chickens was significantly affected by the dietary Nano-Se in comparison to the control group. The apparent digestibility coefficient of EE showed a significant linear increase with increasing Nano-Se level up to 0.4 mg/kg. Meanwhile, all other digestibility coefficients including DM, CP, and CF showed non-significance.

**Table 3:** Effects of Nano-Se on nutrient digestibility of broiler chickens (%).

Items	Se nano particles levels				SEM	p-value	
	0	0.2	0.3	0.4		Linear	Quadratic
Dray matter	75.07	69.70	72.73	70.77	1.513	0.160	0.274
Ether extract	78.58^b^	71.55^b^	87.16^a^	88.35^a^	2.606	0.001	0.130
Crud protein	67.37	60.58	69.35	70.20	1.862	0.971	0.053
Crude fiber	25.20	28.79	25.57	26.03	1.987	0.934	0.441

*SEM* standard error of the means.

^a–^^b^Means within a row with different superscripts are significantly different (p < 0.05).

### Serum biochemical assays

**Figure 2:**
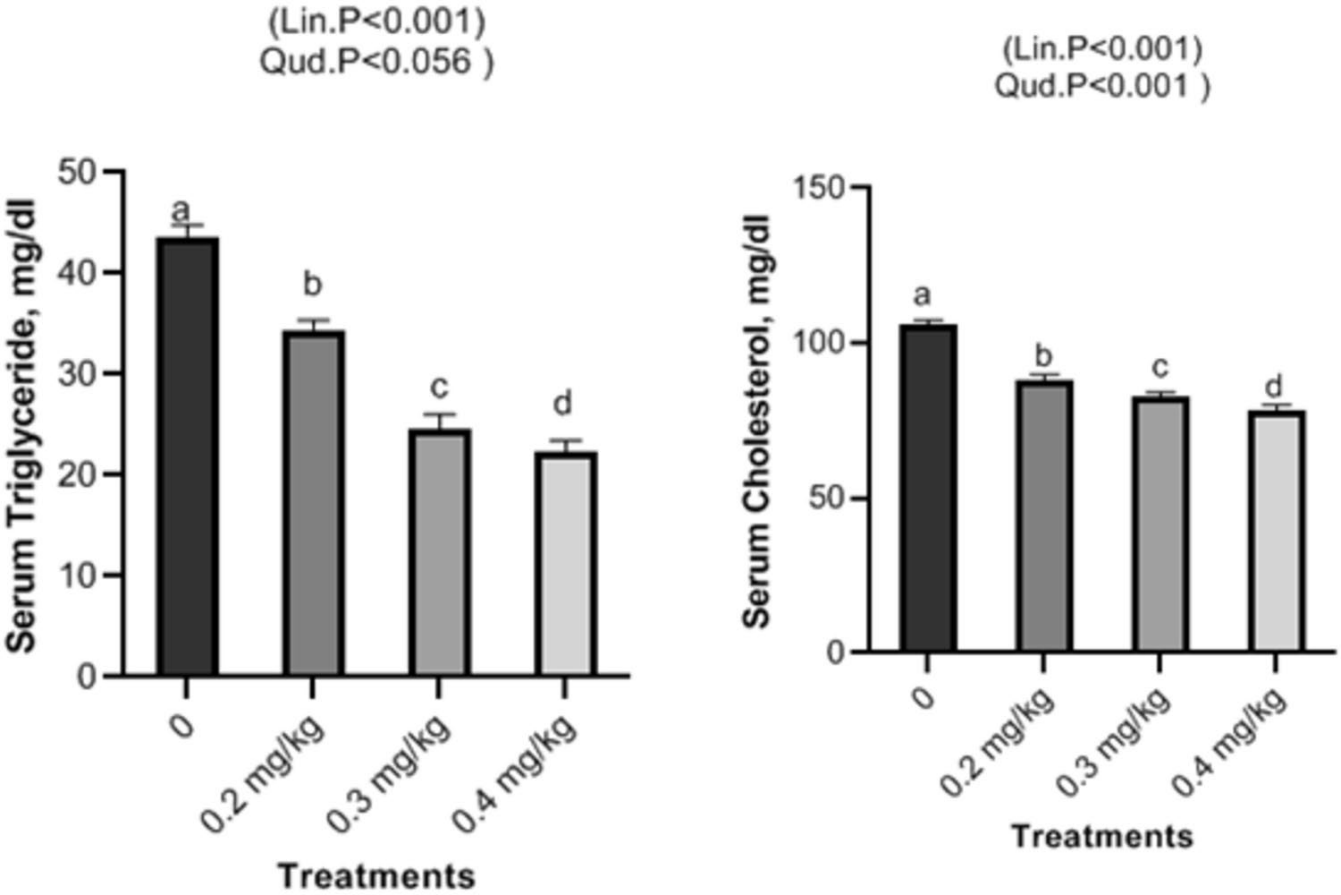
Impact of Nano-Se supplementations on serum triglycerides (mg/dl) and cholesterol (mg/dl) of broiler chickens. The figure’s bars in each column represent the standard error of means, with the birds serving as the experimental unit (n = 48 per treatment). ^a–c^Figures with distinct superscripts exhibit separate columns (p ˂ 0.05).

**Figure 3:**
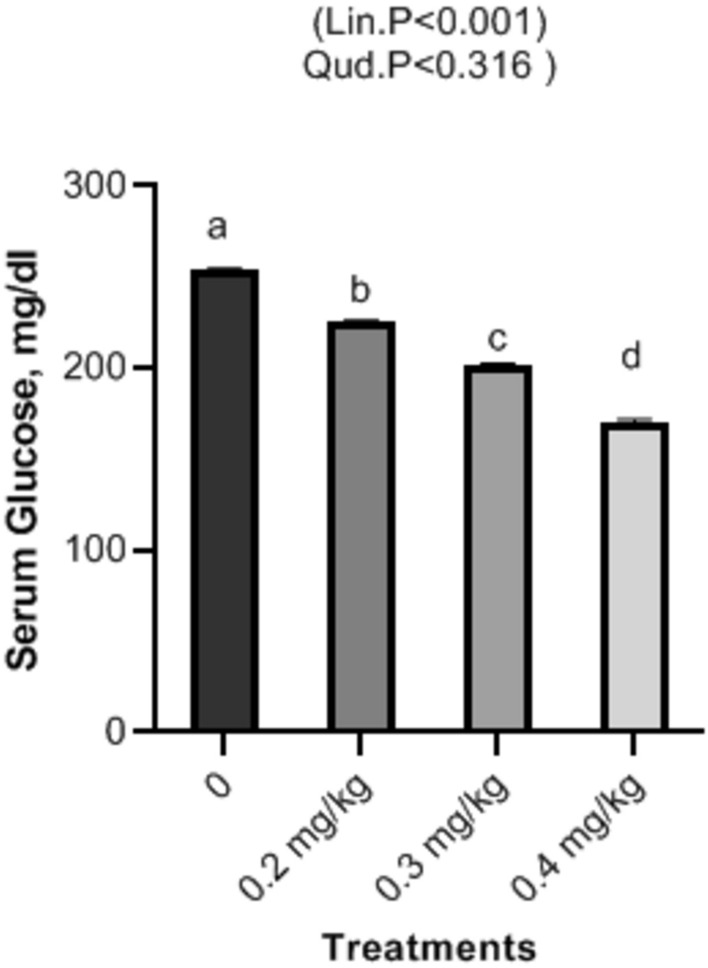
Impact of Nano-Se supplementations on serum glucose (mg/dl) of broiler chickens. The figure’s bars in each column represent the standard error of means, with the birds serving as the experimental unit (n = 48 per treatment). ^a–c^Figures with distinct superscripts exhibit separate columns (p ˂ 0.05).

**Figure 4:**
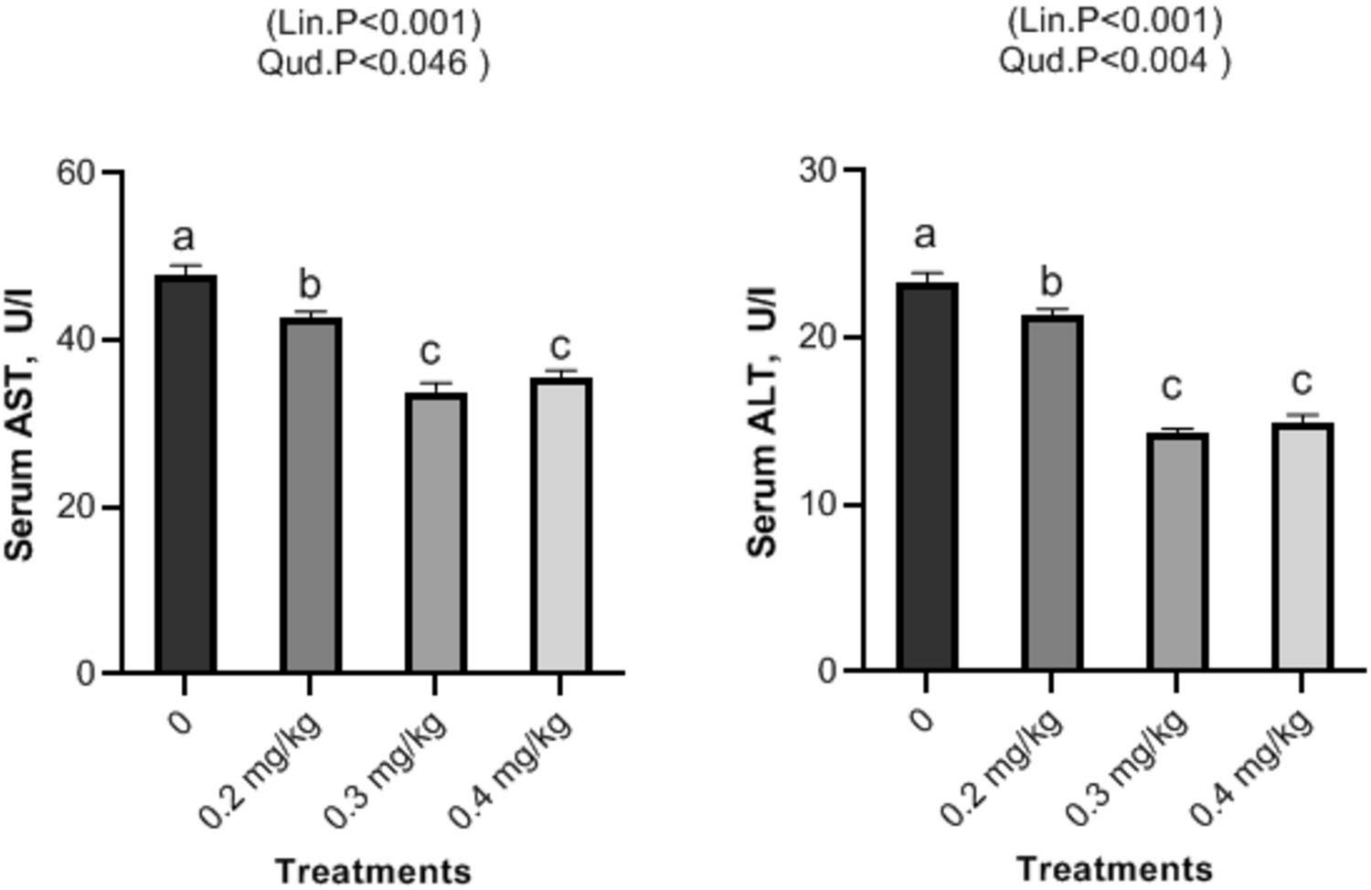
Impact of Nano-Se supplementations on serum alanine aminotransferase (ALT, u/l), aspartate aminotransferase (AST, u/l) enzymes of broiler chickens. The figure’s bars in each column represent the standard error of means, with the birds serving as the experimental unit (n = 48 per treatment). ^a–c^Figures with distinct superscripts exhibit separate columns (p ˂ 0.05).

**Figure 5:**
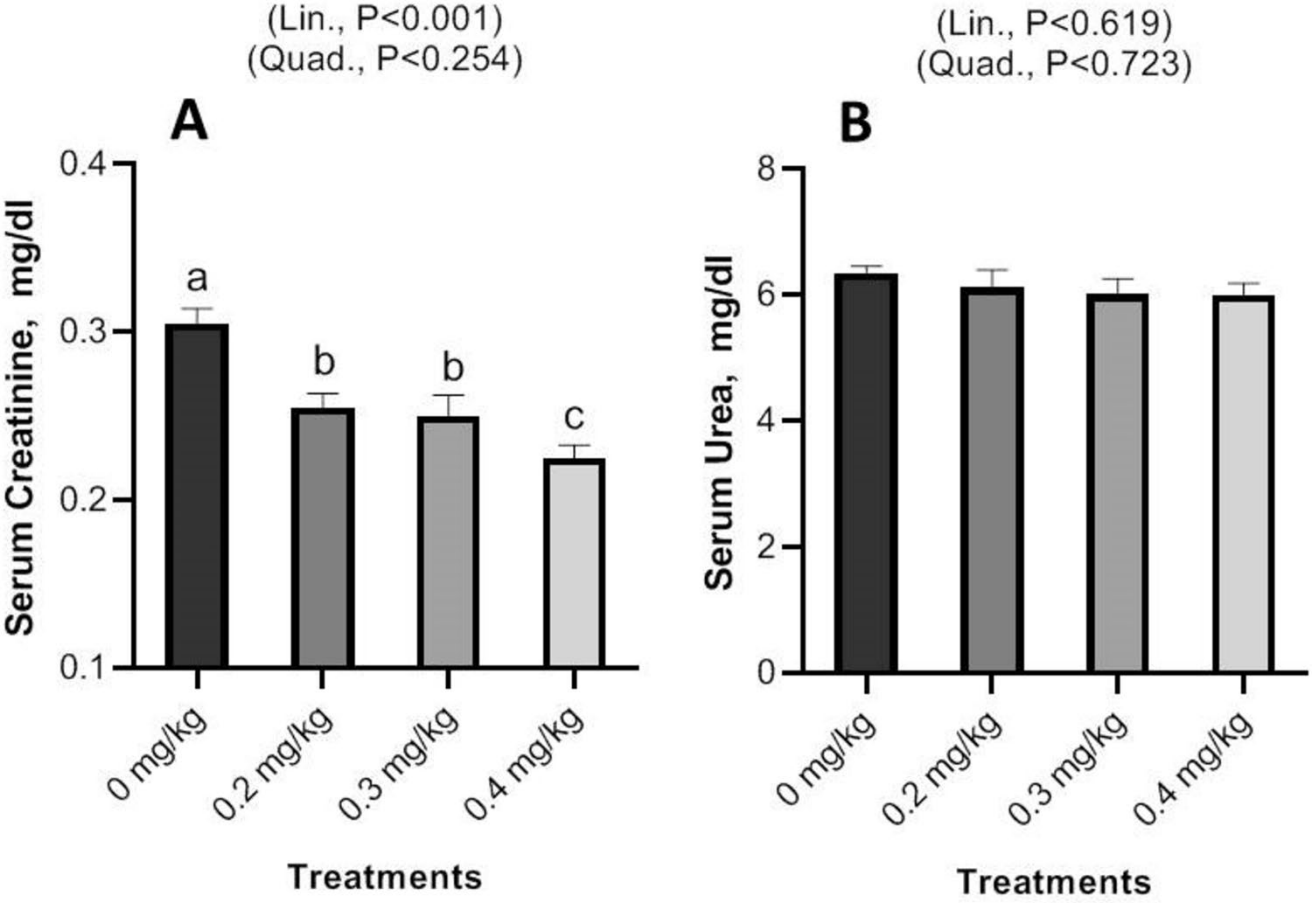
Impact of Nano-Se supplementations on serum creatinine (mg/dl), urea (mg/dl) of broiler chickens. The figure’s bars in each column represent the standard error of means, with the birds serving as the experimental unit (n = 48 per treatment). ^a–c^Figures with distinct superscripts exhibit separate columns (p ˂ 0.05).

**Figure 6:**
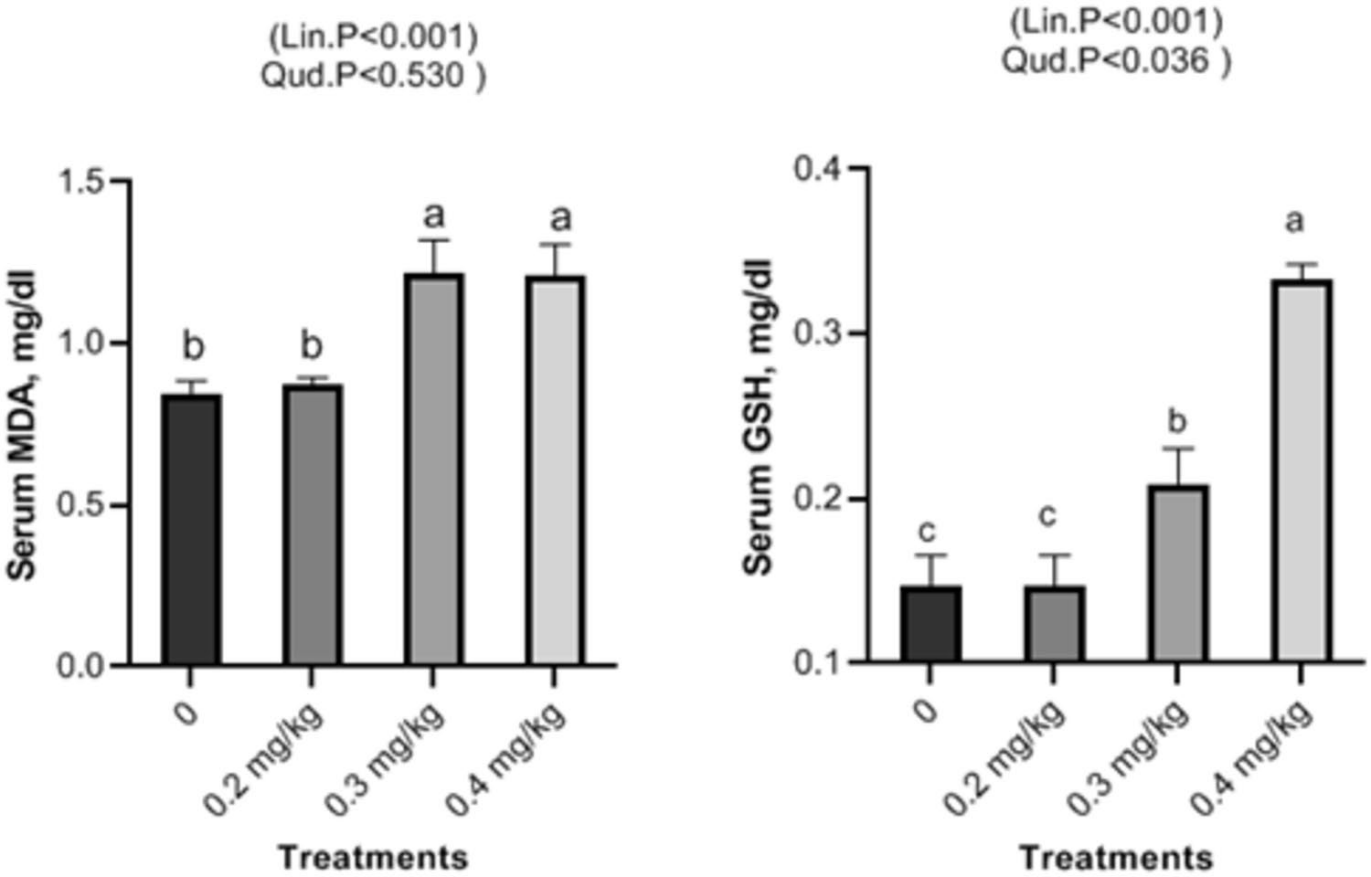
Impact of Nano-Se supplementations on serum malondialdehyde (MDA) and glutathione peroxidase (GSH-Px) activities, of broiler chickens. The figure’s bars in each column represent the standard error of means, with the birds serving as the experimental unit (n = 48 per treatment). ^a–c^Figures with distinct superscripts exhibit separate columns (p ˂ 0.05).

**Figure 7:**
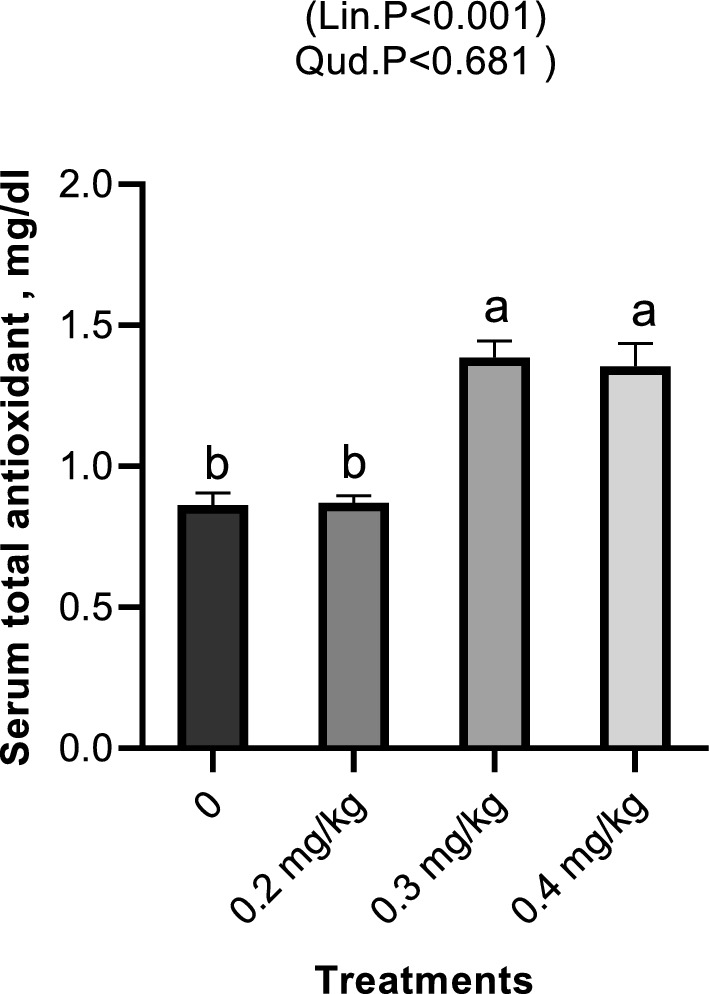
Impact of Nano-Se supplementations on serum total antioxidant (mg/dl) capacity of broiler chickens. The figure’s bars in each column represent the standard error of means, with the birds serving as the experimental unit (n = 48 per treatment). ^a–c^Figures with distinct superscripts exhibit separate columns (p ˂ 0.05).

Dietary Nano-Se revealed a linear and quadratic significant drop in cholesterol and triglycerides levels at 0.2, 0.3, and 0.4 mg/kg, respectively (Fig. 2), but glucose exhibited a linear significant decrease with increasing Nano-Se levels (Fig. 3). Concerning liver function, the ALT, and AST enzymes were significantly decreased with increasing doses of Nano-Se up to 0.3 mg/kg, but 0.4 mg/kg showed significant reductions both linearly and quadratically (Fig. 4). Furthermore, serum kidney function such as creatinine exhibited a linear significant decrease with increasing Nano-Se levels up to 0.4 mg/kg, although urea did not show any significance (Fig. 5). Finally, increasing Nano-Se levels boosted serum Glutathione Peroxidase (GSH-Px) activities linearly and quadratically (Fig. 6). Meanwhile, Malondialdehyde (MDA) significantly decreased with increasing Nano-Se levels up 0.4 mg/kg (Fig. 6). As for the Total Antioxidant (T-AOC) Capacity, a significant linear increase with increasing Nano-Se levels at 0.4 mg/kg was seen when compared to the other levels and the control (Fig. 7).

### Carcass criteria

The carcass criteria of birds as affected by diets supplemented with Nano-Se at 0, 2, 0.3, and 0.4 mg/kg are presented in Table 4. The carcass criteria including dressing, breast, leg, abdominal fat, liver, heart, gizzard, and spleen percentage of broilers did not show any significant differences among Nano-Se supplementation levels.

**Table 4:** Effects of Nano-Se on carcass criteria of broiler chickens.

Items	Se-NPs levels mg/kg				SEM	*p*-value	
	0	0.2	0.3	0.4		Linear	Quadratic
Dressing%	73.54	70.29	74.35	72.38	1.312	0.924	0.629
Breast%	43.70	43.99	46.02	43.11	1.072	0.956	0.143
Leg%	29.84	26.30	28.32	29.26	1.350	0.961	0.104
Liver%	2.156	2.004	2.027	1.955	0.088	0.142	0.684
Heart%	0.541	0.409	0.467	0.430	0.032	0.066	0.156
Gizzard%	1.441	1.464	1.465	1.413	0.076	0.059	0.060
Abdominal Fat%	0.764	0.736	0.719	0.682	0.083	0.663	0.518
Spleen%	0.137	0.125	0.112	0.118	0.009	0.109	0.366

*SEM* standard error of the means.

### Meat quality

The effects of Nano-Se on the physiochemical meat quality of broilers are presented in Table 5. Increasing the level of Nano-Se supplementation resulted in a linear and quadratic significant decrease in cooking loss and a linear significant increase in water holding capacity in the leg; meanwhile, a noticeable linear significant increase in water holding capacity and a linear decrease in cooking loss was also observed in the breast. Furthermore, no significant differences in pH were detected between the treatments in the leg or breast.

**Table 5:** Effects of Nano-Se on physicochemical meat quality of broiler chickens.

Items	Nano-Se levels				SEM	p-value	
	0	0.2	0.3	0.4		Linear	Quadratic
Breast							
pH value	5.066	5.166	5.133	5.200	0.048	0.105	0.733
Cooking loss%	29.27^a^	26.46^bc^	27.74^ab^	25.40^c^	0.736	0.005	0.752
WHC%	24.63^b^	28.05^a^	28.19^a^	29.79^a^	0.597	0.001	0.141
Leg							
pH value	5.131	5.185	5.151	5.183	0.049	0.588	0.829
Cooking loss%	33.90^a^	27.85^b^	27.25^b^	25.52^b^	0.835	0.001	0.017
WHC%	27.05^a^	23.93^b^	25.10^b^	23.44^b^	0.607	0.002	0.244

*SEM* standard error of the means.

^a–^^c^Means within a row with different superscripts are significantly different (p < 0.05).

### Selenium deposition in chicken tissues

**Figure 8:**
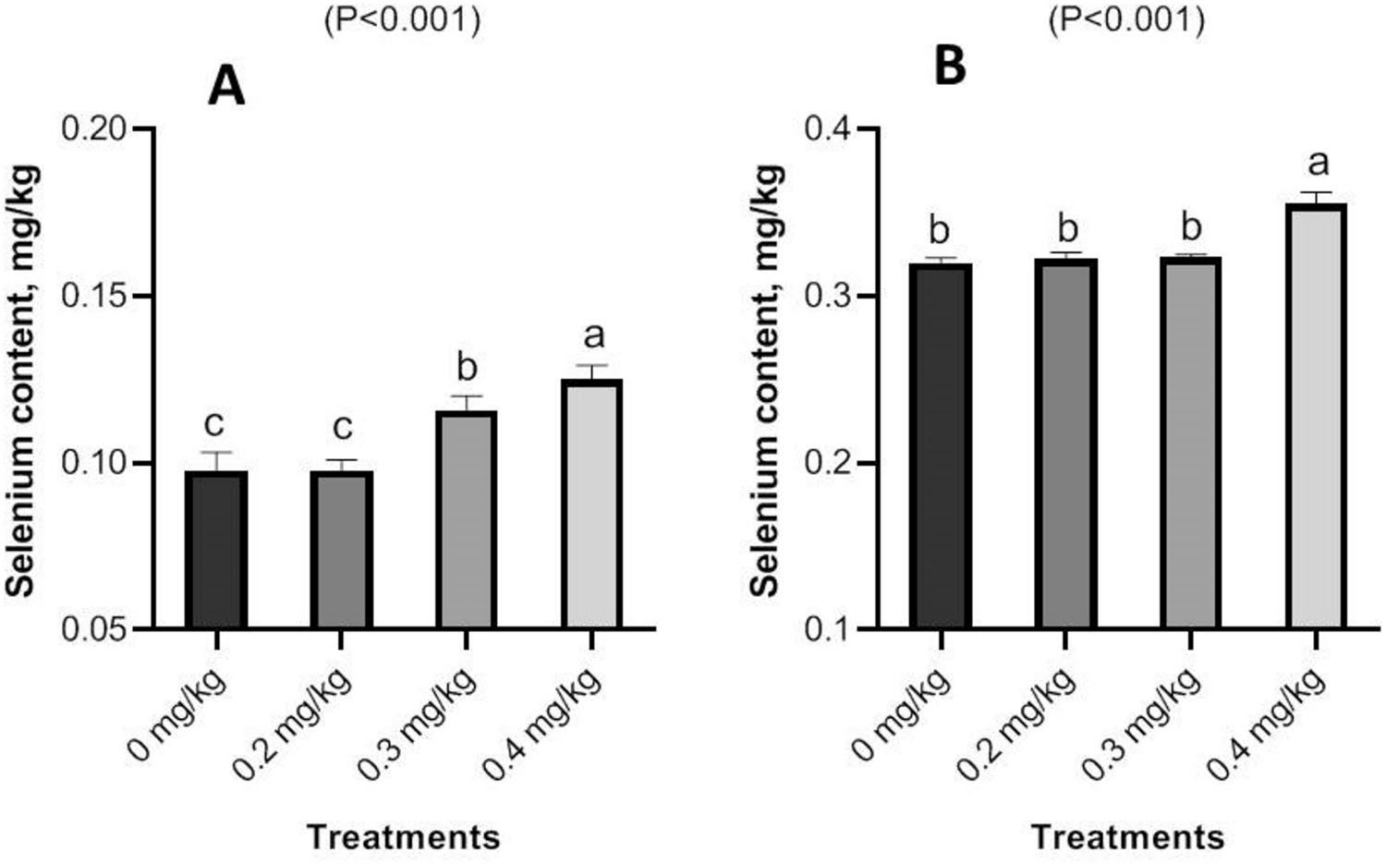
Impact of Nano-Se supplementations on Se content in meat (**A**) and liver (**B**) tissues of broiler chickens. The figure’s bars in each column represent the standard error of means, with the birds serving as the experimental unit (n = 48 per treatment). ^a–c^Figures with distinct superscripts exhibit separate columns (p ˂ 0.05).

Effects of Nano-Se on the deposition of Se in male chickens’ meat and liver tissues are shown in Fig. 8. The broiler diets supplemented with Nano-Se at 0.3 and 0.4 mg/kg showed a significant (p < 0.001) increase in the concentration of Se in the meat when compared to the control groups and the 0.2 mg/kg level. It is also worth noting that the hepatic Se concentration was higher (P < 0.001) at the 0.4 mg/kg supplementation level than at the other levels (0.2 and 0.3 mg/kg) and in the control groups.

### Histopathological findings

**Figure 9:**
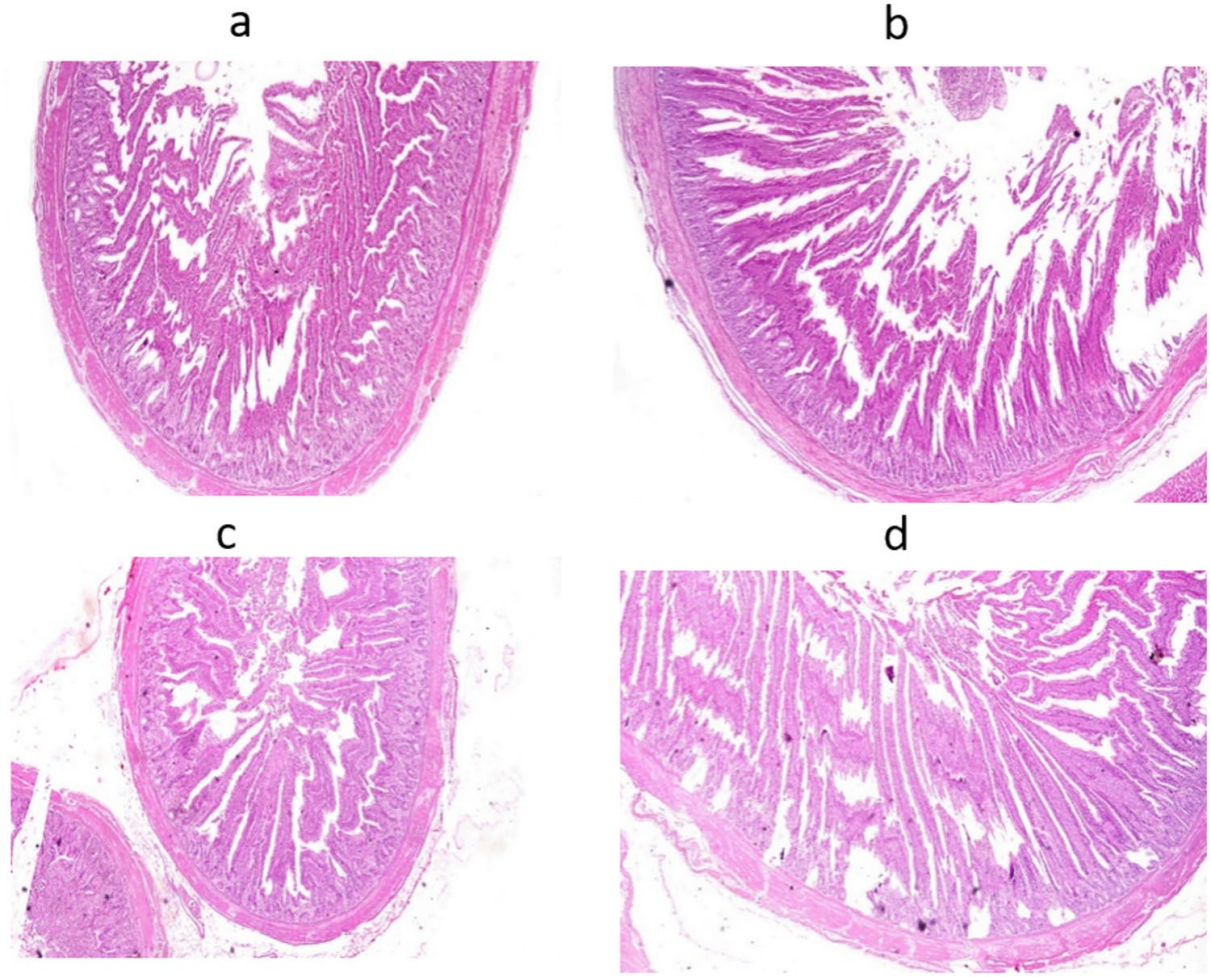
Histopathology of intestine in different groups. (**a**) In control group, (**b**) in Nano-Se 0.2 mg/kg, (**c**) in Nano-Se 0.3 mg/kg, (**d**) in Nano-Se 0.4 mg/kg. Hematoxylin and eosin stain (×40).

**Figure 10:**
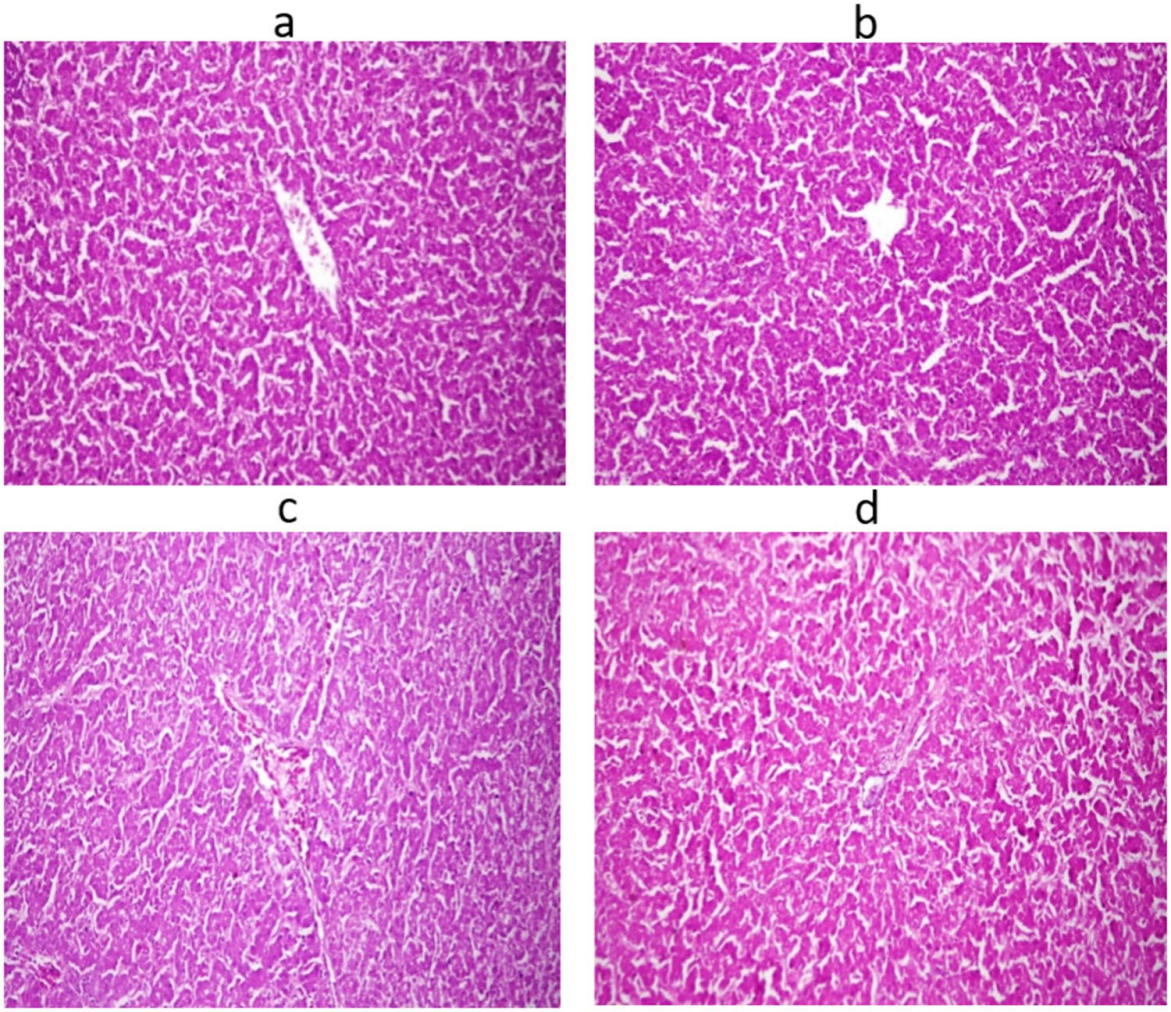
Microscopy of the liver in the control group (**a**) in Nano-Se 0.2 mg/kg, (**b**) in Nano-Se 0.3 mg/kg, (**c**) in Nano-Se 0.4 mg/kg. (**d**) Hematoxylin and eosin stain (×40).

In the control group, microscopy of the intestine indicated modest goblet cell hyperplasia (Fig. 9a). Nano-Se at 0.2 mg/kg supplementation to broiler diets indicated moderate-length intestinal villi in the intestine (Fig. 9b). The intestinal histology in the group fed 0.3 mg/kg Nano-Se showed significant advantages for goblet cell hyperplasia (Fig. 9c). The intestine histology in the group fed 0.4 mg/kg Nano-Se showed lengthy intestinal villi with modest histological change (Fig. 9d). In the control group, liver microscopy revealed normal histological structure (Fig. 10a). Notably, the histopathology of the liver in birds fed Nano-Se at concentrations of 0.2, 0.3, and 0.4 mg/kg exhibited normal histological structure (Fig. 10b–d).

## Discussion

The selenium requirement for Cobb 500 broilers can vary depending on factors such as age, weight, and environmental conditions. However, general guidelines suggest that broiler diets for breeds like the Cobb 500 typically contain selenium at levels ranging from 0.1 to 0.3 mg/kg of feed. Many commercial feed manufacturers follow industry standards and guidelines for selenium supplementation in broiler chicken diets. These standards often align with recommendations provided by organizations such as the NRC^18^. Antinutritional factors in broiler diets, such as phytate, oxalates, tannins, high sulfur content, and mycotoxins, can hinder bulk selenium in feed ingredients absorption. To mitigate this, Nano-Se, a form of selenium with high bioavailability, offers promise. Its nano-sized particles enhance absorption, making it an effective alternative to traditional selenium sources for improved broiler health and performance. Therefore, in this study, we emphasize the positive impact of Nano-Se on growth rate, which directly influences key growth metrics (body weight, body weight gain, and feed conversion ratio) in broiler chickens. The underlying mechanism involves the activation of selenoprotein P and selenoenzyme type I iodothyronine deiodinase. These essential enzymes play a crucial role in thyroid hormone production and selenium transport^33^, potentially contributing to the enhancement of growth performance. Similarly, the elevated levels of thyroid hormone, which regulate the body’s energy metabolism, may underlie our findings of improved growth performance with Nano-Se^34^. The observed enhancements in growth parameters strongly indicate that the improvement is associated with a better FCR. Therefore, the improvements in FCR may stem from Nano-Se’s enhanced role in selenoprotein synthesis, as more than half of these proteins are involved in maintaining redox balance and antioxidant defenses^35^. The foregoing results of the present study sustained by the others indicate that the positive effects of Nano-Se on growth parameters could be attributed to gut microbiota, gut anatomy, and its role in energy metabolism^36^. Moreover, El-Deep et al.^37^ have reported that dietary incorporation of Nano-Se at a concentration of 0.3 mg/kg enhances broiler growth performance and reinforces their immune and antioxidant systems. Our findings corroborate these observations and are consistent with the research conducted by Zhou and Wang^12^, who demonstrated notable improvements in body weight, body weight gain, and feed conversion ratio in broiler groups supplemented with 0.10, 0.30, and 0.50 mg/kg of Nano-Se, respectively. Additionally, Ahmadi et al.^38^ highlighted the positive impact of Nano-Se supplementation at levels ranging from 0.2 to 0.5 mg/kg on BWG and FCR. They identified supplementation with 0.3 to 0.4 mg Nano-Se/kg as the optimal range for enhancing animal productivity while reducing environmental impact. As indicated in the results of the current study, the effects of Nano-Se levels on nutrient digestibility of chickens were nearly at the same level in comparison with the control except for that exhibited significance with the apparent digestibility coefficient of EE of chickens that increased with increasing Nano-Se levels. A notable enhancement in EE digestibility could be attributed to the reduction in plasma cholesterol levels of the birds^39^. Nano-Se may enhance the activity of enzymes involved in lipid digestion and absorption, particularly by promoting the function of lipases and bile salts, which facilitate the breakdown of dietary fats into absorbable forms^40^. In the present study, digestibility values of DM, CP, and CF showed insignificant increases at Nano-Se levels of 0.3 and 0.4 mg/kg. Concerning the various treatment groups, digestibility of DM, CF, and CP at Nano-Se levels of 75, 37.5, 18.75, and 9.375 µg/kg, respectively, were not significant (P > 0.05)^41^. The forgoing results could be interpreted as due to the different surrounding conditions such as climate, experimental time, levels of Se supplementation, the treated species, and balanced diet^42,43^.

In the current study, the carcass criteria including dressing, breast, leg, abdominal fat, heart, liver, spleen, and gizzard of broilers did not show any significant differences among Nano-Se supplementation levels. The lack of significant differences in carcass criteria may be attributed to the use of a well-balanced basal diet. There might be a threshold level beyond which additional Nano-Se does not further enhance carcass characteristics. Consistent with our findings, studies have shown that Nano-Se supplementation at 0.3 or 0.5 mg/kg in feed did not affect carcass traits and organ weights of broiler chickens^44,45^. Similarly, supplementation with Nano-Se at levels of 50, 150, and 300 µg/kg did not result in significant differences in carcass criteria^46^. In the present study, Nano-Se supplementation in broiler diets leads to remarkable improvements in both cooking loss and water-holding capacity without effects on the pH value of meat. Nano-Se supplementation reduces water leakage (drip) during meat cooking by maintaining cell membrane integrity in muscle fibers^47^. This reduction in drip loss immediately improves the meat’s water-holding capacity. This effect is attributed to reduced drip loss, pH regulation, antioxidant properties, and maintenance of protein structure^48,49^. As a result, broiler meat retains more water during cooking, enhancing its overall quality. Additionally, because the cell membranes were more intact when birds were supplemented with Nano-Se at a dose of 0.3 mg/kg, there was less drip loss^9^.

In this investigation, Nano-Se dietary supplementation shows a significant decrease respectively at levels of 0.2, 0.3, and 0.4 mg/kg in cholesterol, and triglycerides and with rising selenium levels of broilers. Selenium is involved in the regulation of gene expression, including genes related to lipid metabolism. By influencing the expression of genes involved in cholesterol and triglyceride metabolism, selenium may help modulate lipid levels in the blood. Nano-Se exerts its influence on cholesterol receptor synthesis and the function of HMG-Coenzyme, a pivotal regulator of blood lipid levels^50^. These combined effects contribute to lowering blood cholesterol levels. Nano-Se’s diverse mechanisms support lipid balance, antioxidant defense, and thyroid function, resulting in enhanced serum cholesterol and triglyceride levels in broilers^51^. Our findings are consistent with previous research, particularly the study by Mohapatra et al.^52^, which demonstrated that chicks supplemented with 0.3 mg/kg of Nano-Se experienced significantly reduced triglyceride and cholesterol levels compared to the control group. Likewise, dietary Nano-Se at 0.5 mg/kg led to noteworthy reductions in plasma triglycerides and total cholesterol in broiler diets, as reported by Saleh and Ebeid^53^. Meanwhile, concerning liver function i.e. ALT and AST, as well as serum kidney function i.e. creatinine significant decrease was observed in all treatments with increasing levels of Nano-Se. The multifaceted effects of Nano-Se, encompassing antioxidant properties, metabolic regulation, modulation of gene expression, and activation of the AMPK pathway, collectively contribute to the enhanced liver and kidney functions observed in broilers^54^. Furthermore, Nano-Se significantly reduced AST, ALT, triglycerides, and cholesterol levels in broilers compared to the control group^55^. Similarly, Nano-Se markedly improved liver and renal functions in rabbits compared to the control group^56^. It is noteworthy that liver oxidative damage can be assessed through ALT and AST enzyme levels. In this study, serum levels of cholesterol, creatinine, ALT, and AST in broiler chickens were significantly reduced by dietary supplementation with Nano-Se at doses of 0.2, 0.3, and 0.4 mg/kg. These results were supported by findings obtained by Ref.^57^ who reported the reduction of AST, ALT, and creatinine levels in sera of Cobb broilers fed Nano-Se at concentrations of (0.15 ppm), (0.075 ppm) and (0.0375 ppm), respectively. At this end, the significant decrease in both ALT and AST could be attributed to including Nano-Se in the diet of broilers and deciphered as due to Nano-Se effect on the thyroid (T3) hormone’s dominant effects on fat metabolism^58^. Furthermore, the present study revealed a significant increase in the antioxidant serum levels of GSH and T-AOC, accompanied by a significant decrease in MDA levels with increasing Nano-Se concentrations. Nano-selenium is widely recognized as an effective antioxidant that enhances serum health indicators^59^. Previous studies have reported significant improvements in glutathione peroxidase activities (GSH-Px) when birds were supplemented with Nano-Se at doses ranging from 0.2 to 0.5 mg/kg^60^. Additionally, supplementation with 0.3 mg/kg of Nano-Se has been shown to enhance glutathione peroxidase activities in serum, liver, and muscle^9^. Moreover, broilers fed Nano-Se-supplemented diets exhibited increased GSHPx activity and decreased MDA levels compared to the control group^61^. The elevation in broilers’ antioxidant status can be credited to the favorable outcomes resulting from dietary supplementation with 0.3 mg/kg of Nano-Se^62^. Additionally, selenium supplementation into animal diets has demonstrated the ability to elevate the body’s glutathione pool and Se-containing antioxidant enzymes^63^. This augmentation in antioxidant status has been corroborated by several studies emphasizing selenium’s role in activating GSH-Px^64^. Notably, in both broilers and layers, the inclusion of Nano-Se in feed formulations has been associated with heightened antioxidant status and increased glutathione peroxidase activity^65^. Herein, our results exhibit a significant increase in GPX in supplemented Nano-Se groups as compared with the control. Compared to the control group and other treatment groups, birds fed with 0.1875 mg/kg of selenium nanoparticles showed increased glutathione peroxidase cellular activity^66^. Additionally, compared to the control group, there was an increase in glutathione peroxidase activity in both serum and tissue in the groups treated with Nano-Se^10^. Comparing the vaccinated group supplemented with Nano-Se (0.15 ppm) to the same concentration of sodium selenite (0.15 ppm), serum levels of MDA were significantly lower^67^. Additionally, broiler diets containing Nano-Se not only reduced MDA levels in the muscles and liver but also enhanced the functions of GSHPx^68^. The observed decrease in MDA can be attributed to Nano-Se’s impact on antioxidant enzymes responsible for neutralizing reactive oxygen species^69^. In line with the research by Boostani et al.^3^, selenium plays an active role in antioxidant defense mechanisms. As an essential constituent of the enzyme selenium-dependent glutathione peroxidase, selenium efficiently diminishes peroxide levels and protects cells.

Selenium stands as a vital element in poultry nutrition, playing a multifaceted role in supporting various physiological functions essential for bird growth and health maintenance^70^. Its significance extends beyond mere sustenance, as it actively contributes to the nutritional value and metabolic processes of feed, thereby exerting a profound influence on overall growth performance^71^. Notably, Nano-Se has garnered considerable attention owing to its remarkable attributes, including potent absorption capabilities, heightened catalytic efficiency, pronounced surface activity, and comparatively low toxicity when compared to other chemical Se forms^72^. Of particular importance is its impressive bioavailability, facilitating efficient absorption from the intestinal lumen into the avian body^73^. Furthermore, Se emerges as a potent agent in combating oxidative stress, boasting antioxidant properties that aid in safeguarding cellular integrity and mitigating the detrimental effects of free radicals. Its potential extends even further, with documented anticancer, antibacterial, and antiprotozoal properties, underscoring its multifaceted benefits in promoting poultry health and well-being^72–75^. As research continues to unveil the diverse roles and benefits of Se in avian nutrition, its integration into poultry diets remains pivotal for optimizing growth, health, and overall production efficiency.

Further studies are necessary to elucidate the mode of action of Nano-Se and its impact on antioxidant activities.

Groups supplemented with Nano-Se at 0.3 and 0.4 mg/kg exhibited a significantly higher increase in selenium concentration in meat compared to the 0.2 mg/kg level and the control groups. The findings indicate that as the concentration of Nano-Se added to the feed increases, there is a corresponding increase in tissue deposition. This phenomenon may be attributed to Nano-Se’s ability to efficiently saturate selenium, thereby enhancing its retention, as well as the increased absorption of selenium from the mucous membrane of the small intestine, leading to higher tissue deposition^76^. Moreover, incorporating Nano-Se into broiler diets resulted in a significant elevation in selenium concentration in the breast muscles of the chickens compared to the control group^77^. Nano-Se levels ranging from 0.1 to 0.3 mg/kg demonstrated the highest selenium retention in both breast and thigh muscles^57^. Additionally, supplementation of diets with Nano-Se at 0.30 mg/kg effectively increased the selenium content of tissues^9^. An interpretation of the observed concentration behavior in tissues suggests that supplementing diets with Nano-Se particles, which possess novel transport and uptake characteristics, leads to greater absorption efficiencies. Consequently, this may result in higher Nano-Se retention within the tissues^78^. In the current study, a microscopic review of the intestinal samples in the studied groups showed some histopathological changes secondary to increasing Nano-Se levels in supplemented diets. These gradual changes in the form of goblet cell hyperplasia from mild to moderate, followed by changes in the length of intestinal villi, finally end up with mild histopathological alteration. Additionally, Microscopy of the liver in the control group revealed normal histological structure. Similarly, the histopathology of the liver in the examined groups also revealed normal histological structure. In comparison to a normal view, the broilers supplemented with 0.5 mL Nano-Se show preservation of the intestinal villi, glands, and muscular layer with only minor villi lining epithelium loss^79^. However, in contrast to the unsupplemented control group, histopathological photomicrographs of the liver in broilers supplemented with Nano-Se (at doses of 0.2, 0.3, and 0.4 mg/kg) revealed improved and nearly normal tissue architecture^74^. Feed supplementation is an intriguing domain in poultry nutrition, especially with the emergence of novel feeding strategies^80–83^. This observed improvement closely resembled findings from Alkhudhayri et al.^84^, who demonstrated enhanced histological tissue architecture following Nano-Se supplementation. Further studies are required to elucidate the mode of action of Nano-Se and its impact on the histopathological effects of broilers’ internal organs, in order to provide a complete understanding of the Nano-Se pathway in tissues.

## Conclusions

In conclusion, nutritional supplementation with Nano-Se up to 0.4 mg/kg improved growth performance, nutrient digestibility, meat quality, and antioxidant activity of broiler chickens. Additionally, dietary Nano-Se supplementation revealed beneficial hypolipidemic effects by lowering serum cholesterol and triglyceride levels. Nano-Se exhibited anti-inflammation properties that protect liver tissues, as evidenced by normal tissue structure and liver enzymes. Overall, the best performance of broilers was observed with Nano-Se supplementation at 0.3 mg/kg, highlighting its potential as a novel supplement for broiler diets. Additional research is needed to clarify the mechanism of action of Nano-Se and its influence on the sustainability of productive performance, as well as its role in antioxidants, antimicrobial activities, immunity-related genes, and histopathological effects on broilers’ internal organs. This is essential for a comprehensive understanding of the Nano-Se pathway in broiler chickens feeding.

## Data Availability

The datasets used and/or analyzed during the current study are available from the corresponding author upon reasonable request.
